# Supplementation with Sucrosomial® iron leads to favourable changes in the intestinal microbiome when compared to ferrous sulfate in mice

**DOI:** 10.1007/s10534-021-00348-3

**Published:** 2021-10-25

**Authors:** Martha Zakrzewski, Sarah J. Wilkins, Sheridan L. Helman, Elisa Brilli, Germano Tarantino, Gregory J. Anderson, David M. Frazer

**Affiliations:** 1 Medical Genomics, QIMR Berghofer Medical Research Institute, Herston, Australia; 2 Iron Metabolism Laboratory, QIMR Berghofer Medical Research Institute, Herston, Australia; 3 Molecular Nutrition Laboratory, QIMR Berghofer Medical Research Institute, Royal Brisbane Hospital, Locked Bag 2000, Herston, QLD 4029 Australia; 4 Faculty of Medicine, The University of Queensland, St Lucia, Australia; 5 R&D Department, PharmaNutra S.p.A, Pisa, Italy; 6 School of Chemistry and Molecular Bioscience, The University of Queensland, St Lucia, Australia; 7 School of Biomedical Sciences, The University of Queensland, St Lucia, Australia; 8 School of Biomedical Sciences, The Queensland University of Technology, Gardens Point, Brisbane, Australia

**Keywords:** Sucrosomial® iron, Ferrous sulfate, Iron supplementation, Intestinal microbiome, Iron deficiency

## Abstract

Iron deficiency is one of the most common nutritional deficiencies worldwide and is often treated with oral iron supplements. However, commonly used supplements, including those based on ferrous iron salts, are associated with gastrointestinal side effects and unfavorable changes in the intestinal microbiome. Sucrosomial® iron is a novel iron formulation that is effective at treating iron deficiency, and with fewer gastrointestinal side effects, yet its effect on the gut microbiome has not been examined previously. Thus, we treated mice for two weeks with diets containing either Sucrosomial® iron or ferrous sulfate as the sole iron source and examined bacterial communities in the intestine using 16S Microbial Profiling of DNA extracted from feces collected both prior to and following dietary treatment. Mice treated with Sucrosomial® iron showed an increase in Shannon diversity over the course of the study. This was associated with a decrease in the abundance of the phylum Proteobacteria, which contains many pathogenic species, and an increase in short chain fatty acid producing bacteria such as *Lachnospiraceae*, *Oscillibacter* and *Faecalibaculum*. None of these changes were observed in mice treated with ferrous sulfate. These results suggest that Sucrosomial® iron may have a beneficial effect on the intestinal microbiome when compared to ferrous sulfate and that this form of iron is a promising alternative to ferrous iron salts for the treatment of iron deficiency.

**Supplementary Information:**

The online version contains supplementary material available at 10.1007/s10534-021-00348-3.

## Introduction

Iron deficiency, and its more severe manifestation iron deficiency anaemia (IDA), are major global health issues estimated to affect more than two billion people worldwide (Camaschella 2019). They are most common in young children and women of child-bearing age (Pasricha et al. 2010; Zimmermann and Hurrell 2007), and occur because the body cannot absorb enough iron from the diet to meet body requirements or replenish iron losses. Iron deficiency is often multifactorial, with the most common causes being insufficient dietary intake, the rapid growth that occurs during infancy, and blood loss through menstruation or gastrointestinal bleeding (Pasricha et al. 2010; Zimmermann and Hurrell 2007). Iron deficiency, and particularly IDA, can lead to impaired cognitive development in children, and diminished work capacity and mental function in adults (Pasricha et al. 2010). IDA in pregnant women is associated with low birth weight, prematurity and maternal morbidity (Breymann 2015; Milman et al. 2016).

Currently, the most widely used and most cost-effective treatment for iron deficiency and IDA is oral supplementation with ferrous iron salts such as ferrous sulfate and ferrous gluconate. Although ferrous iron is highly bioavailable, a large proportion of the iron from oral supplements is not absorbed and remains in the intestinal lumen, where it can catalyse the formation of reactive oxygen species (Lund et al. 1999; Orozco et al. 2012). This can cause oxidative damage to the intestinal mucosa (Kaye et al. 2008), and is thought to cause many of the gastrointestinal side effects associated with oral iron supplements, such as nausea and abdominal pain, and this in turn results in non-adherence to treatment in up to 50% of patients (Tolkien et al. 2015).

The iron remaining in the lumen of the intestine following supplementation can also impact the intestinal microbiome. Several studies show an increase in the abundance of pathogenic enterobacteria and, in some cases, fecal calprotectin, a marker of intestinal inflammation, in infants supplemented with iron (Jaeggi et al. 2015; Simonyte Sjodin et al. 2019; Zimmermann et al. 2010). A decrease in the abundance of beneficial bacteria, such as *Bifidobacterium* and *Lactobacillus* species, has also been observed in the gut of iron deficient infants receiving iron supplements (Jaeggi et al. 2015; Paganini et al. 2017). While the health implications of these changes in the absence of intestinal pathogens are not known, the widespread use of iron supplements during pregnancy and infancy, and the growing appreciation of the importance of the intestinal microbiome in many aspects of development, including the correct functioning of both the immune and central nervous systems (Sharon et al. 2016; Tamburini et al. 2016), suggest that such changes could be a cause for concern. Conversely, iron supplements that have negligible, or even positive, impacts on the microbial communities in the intestine, in addition to being an effective source of iron, could be of major benefit to those suffering from iron deficiency, particularly during periods of rapid growth and development.

Sucrosomial® iron is a relatively new iron supplement consisting of a ferric pyrophosphate core surrounded by a phospholipid matrix that contains sucrester, a surfactant with absorption enhancing properties (Gomez-Ramirez et al. 2018). This form of iron is effective for treating iron deficiency of varying etiology (Elli et al. 2018; Mafodda et al. 2017; Parisi et al. 2017; Pisani et al. 2015), and there is evidence suggesting that it has fewer side effects than supplements based on ferrous iron (Abbati et al. 2019; Elli et al. 2018). While the precise mechanism of Sucrosomial® iron absorption by the intestine has yet to be determined, in vivo studies in both humans and animal models suggest that Sucrosomial® iron is at least as well absorbed as ferrous iron salts (Asperti et al. 2018), with some studies indicating greater bioavailability (Mafodda et al. 2017; Parisi et al. 2017). However, the effect of this supplement on the microbial communities in the gastrointestinal tract has not been previously examined. In this study, we have used the mouse as a model to compare the effects of Sucrosomial® iron with ferrous sulfate, a commonly used iron supplement, on the intestinal microbiome during iron deficiency.

## Materials and methods

### Animals

All experiments were performed on male C57BL/6 mice that were bred in the QIMR Berghofer Animal Facility and were maintained on a standard rodent pellet diet (120 mg/kg iron; Norco Stockfeed, Lismore, Australia) unless otherwise indicated. Animals were housed in an Optimice IVC rack system (Animal Care Systems, Dromana, Australia) on a 12-h light/dark cycle (light between 8 AM and 8 PM local time) with unlimited access to food and water. Each cage contained PuraChip Aspen Enrichment Bedding (Able Scientific, Perth, WA, Australia), with unscented facial tissues to provide environmental enrichment. Four week old mice were switched to an iron deficient diet based on AIN93G (~ 1 mg/kg iron; Specialty Feeds, Glen Forrest, Australia) and maintained on this diet for two weeks. At six weeks of age (i.e. two weeks on the deficient diet), feces were collected, snap frozen and stored at − 80 °C for later microbiome analysis. This was the control sample for each mouse and represents the baseline microbiome of that particular animal when the intestinal lumen is iron deficient. Following fecal collection, the mice were switched to the same base diet containing 50 mg/kg iron as either ferrous sulfate (Sigma-Aldrich, North Ryde, Australia) or Sucrosomial® iron (PharmaNutra S.p.A., Pisa, Italy). A separate cohort of mice remained on the iron deficient diet as a control group. At eight weeks of age (i.e. two weeks on the iron containing diets), fecal samples were again collected from each mouse. The mice were then anesthetized (200 mg/kg ketamine and 10 mg/kg xylazine) and blood collected by cardiac puncture prior to euthanasia by cervical dislocation. Liver tissue was collected for subsequent iron analysis.

### Hematological parameters and iron status markers

Hematological parameters were assessed immediately after euthanasia using a Coulter Ac·T diff Hematology Analyzer (Beckman Coulter, Gladesville, Australia). Serum iron parameters were determined using the Iron/TIBC Reagent Kit (Pointe Scientific, Canton, MI) as previously described (Frazer et al. 2017). Tissue iron levels were assayed by colorimetric assay as previously described (Torrance and Bothwell 1968).

### Statistical analysis for iron parameters

All experimental groups contained ten mice. For the analysis of iron and hematological parameters, statistical differences between groups were calculated using ANOVA followed by Tukey’s post hoc testing for groups with equal variance or Games-Howell post-hoc testing for those with unequal variance. Significant differences in variance were identified using Levene’s test. A *P* value of less that 0.05 was considered statistically significant.

### DNA extraction and 16S rRNA amplification and sequencing

DNA extraction and amplicon sequencing were performed at the Australian Genome Research Facility. DNA was extracted from up to 50 mg of faecal sample using the DNeasy PowerSoil Pro Kit (QIAGEN). The 16S V1–V3 region was PCR-amplified using the 27F (AGAGTTTGATCMTGGCTCAG) and 519R (GWATTACCGCGGCKGCTG) primer set. The samples were pooled and sequenced on the Illumina MiSeq platform utilising illumina’s XT Indexes and Paired End Sequencing chemistry. Raw sequencing data files have been uploaded to the NCBI Sequence Read Archive at http://www.ncbi.nlm.nih.gov/bioproject/754104 with the project accession number PRJNA754104.

### Data processing and analysis

Raw data were quality controlled and joined using PEAR (v0.9.6). Primer sequences were removed using Cutadapt (v1.13). High quality reads were imported to QIIME2 and processed using the paired-multiplexed pipeline DADA2 to remove chimeras and form ASV. ASVs were classified using the SILVA (v138) dataset as a reference. Unifrac distances were calculated based on the ASVs. Data were analysed using Calypso. Principal coordinates analysis (PCoA) was used to ordinate the beta diversity (overall microbial community between samples) of the gut microbiota using the unweighted UniFrac matrix in a two-dimensional space. The permutational multivariate ANOVA (ADONIS) with 999 permutations of the unweighted UniFrac matrix was applied to detect associations between the variation in the gut microbiome, and diet and cage effects. ADONIS was carried out twice by changing the order of the factors diet type and cage ID. Hierarchical cluster analysis was applied to visualize the relationships of the samples according to diet, mouse ID and cage number. Changes in alpha diversity (overall microbial community within samples) were measured using mixed effect regression analysis with Shannon diversity/richness indices as the outcome, mouse and cage as random effects, diet as the independent variable and time as a fixed effect. Mixed effects regression analysis was repeated using taxon abundances as outcome to identify associations between taxa and diet by incorporating random effects from cages and mouse ID. *P* values were adjusted using the Bonferroni correction to avoid false positives. Correlation was measured between iron indices and Shannon diversity or richness using Pearson correlation.

## Results

### Sucrosomial® iron is equally as effective as ferrous sulfate in overcoming iron deficiency

**Fig. 1:**
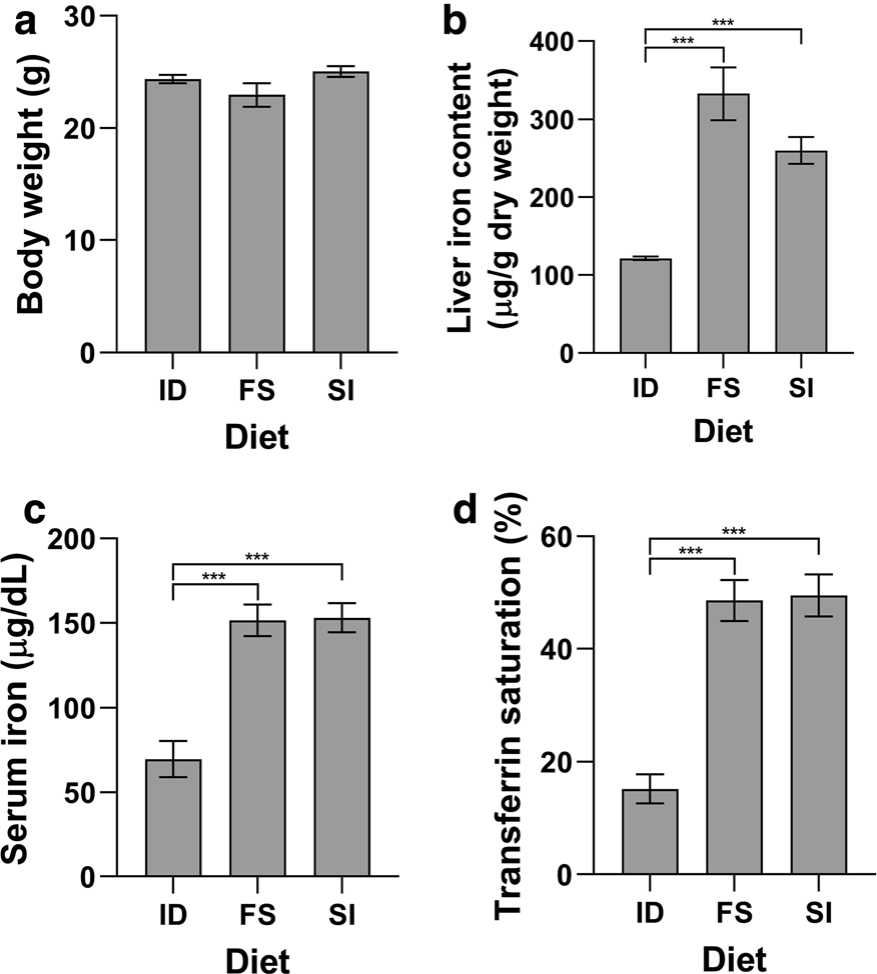
Iron status markers from mice fed Sucrosomial® iron or ferrous sulfate. Mice were fed an iron deficient diet for two weeks prior to being switched to a diet containing 50 mg/kg iron as either Sucrosomial® iron (SI) or ferrous sulfate (FS) for a further two weeks prior to sacrifice. A control group remained on the iron deficient diet for the second two week period (ID). Body weight **a** was recorded and liver iron concentration **b**, serum iron **c** and transferrin saturation **d** were determined. All data represent mean ± SEM with 10 mice per group. ***P < 0.005

**Fig. 2:**
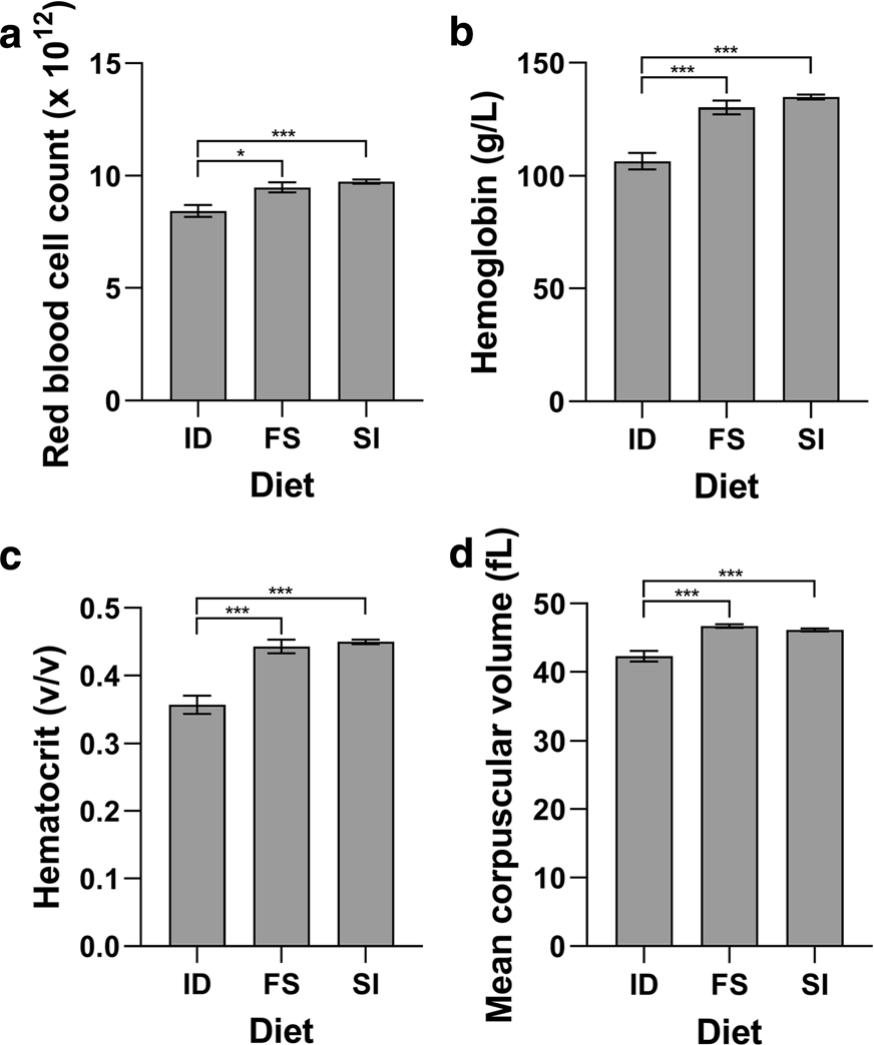
Hematological parameters from mice fed Sucrosomial® iron or ferrous sulfate. Mice were fed an iron deficient diet for two weeks prior to being switched to a diet containing 50 mg/kg iron as either Sucrosomial® iron (SI) or ferrous sulfate (FS) for a further two weeks prior to sacrifice. A control group remained on the iron deficient diet for the second two week period (ID). Red blood cell count **a**, hemoglobin **b**, hematocrit **c** and mean corpuscular volume **d** were determined. All data represent mean ± SEM with 10 mice per group. *P < 0.05, ***P < 0.005

Four week old male C57BL/6 mice were fed an iron deficient diet for two weeks prior to being switched to a diet containing 50 mg/kg iron as either Sucrosomial® iron or ferrous sulfate for a further two weeks. Another group of mice remained on the iron deficient diet for the entire four week period. No significant differences in body weight were recorded between all three groups (Fig. 1a). To ensure that the intestinal microbiome was not affected by differences in systemic iron status in either group fed the iron containing diets, we examined liver and serum iron levels as well as haematological parameters. Both Sucrosomial® iron and ferrous sulfate were able to overcome the effects of iron deficiency as indicated by significant increases in liver iron concentration, serum iron levels, transferrin saturation, red blood cell count, hemoglobin, hematocrit and mean corpuscular volume (Figs. 1a–d, 2a–d). No significant difference was observed between the Sucrosomial® iron and ferrous sulfate treated groups for any of these parameters.

### Mice fed a Sucrosomial® iron containing diet exhibit increased microbial diversity

**Fig. 3:**
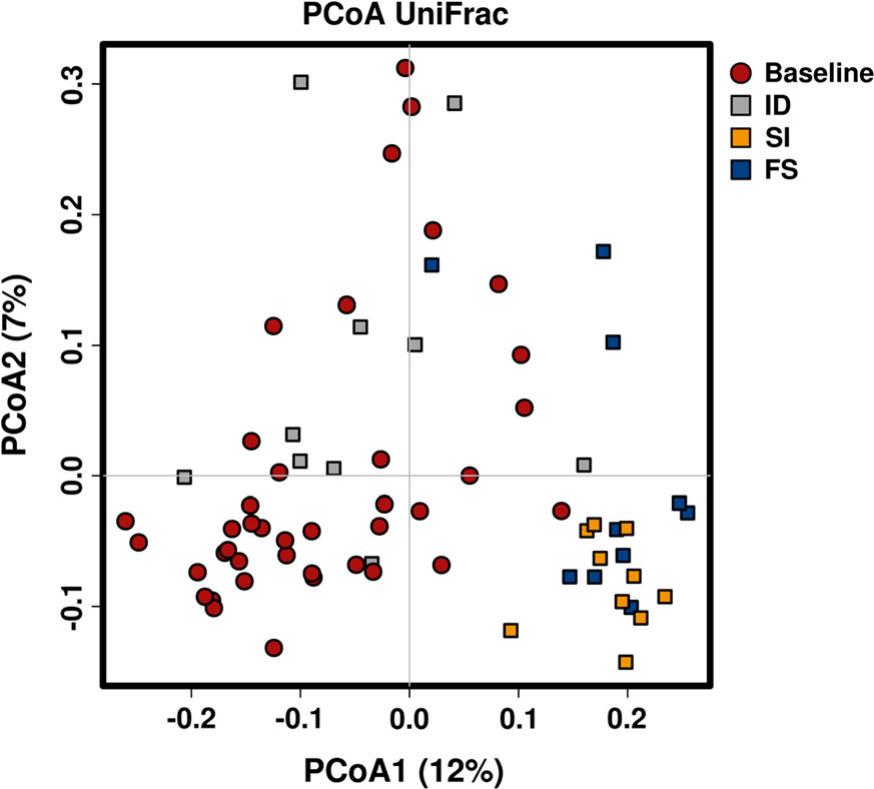
PCoA plot of the unifrac distance among the baseline, iron-deficient and iron-rich diets. Gut microbiota was assessed in mice at baseline (red, circle) and after two weeks on an iron-deficient (ID, gray square), Sucrosomial® iron (SI, yellow square) or ferrous sulfate (FS, blue square) diet. Each data point represents a sample. The distance between each sample indicates the dissimilarity between the microbial overall composition. The x-axis describes 12% of the microbial variation and tends to separate mice fed iron-deficient from iron-rich diets

Faecal samples for microbiome analysis were collected from all mice, both after the initial two weeks on the iron deficient diet (baseline) and after a further two weeks on either iron containing diet. The control group that remained on the iron deficient diet was sampled at two and four weeks. Beta diversity analysis was carried out to identify differences in overall microbial composition between the different diets/time points. PCoA shows that samples taken from mice fed either of the iron rich diets largely clustered together and were able to be distinguished from the samples taken from mice on iron deficient diets and at baseline (Fig. 3). ADONIS analysis confirmed that both diet and cage have an impact on the overall microbial composition (diet, ADONIS, *P* < 0.005; cage, ADONIS, *P* < 0.005). It is frequently the case in animal studies that mice housed in the same cage have more similar microbial profiles than those in different cages. (Hildebrand et al. 2013) After correcting for any cage effect, the effect of diet remained. Hierarchical clustering confirmed a grouping of the samples by diet and cage (Supplementary Fig. S1).

**Fig. 4:**
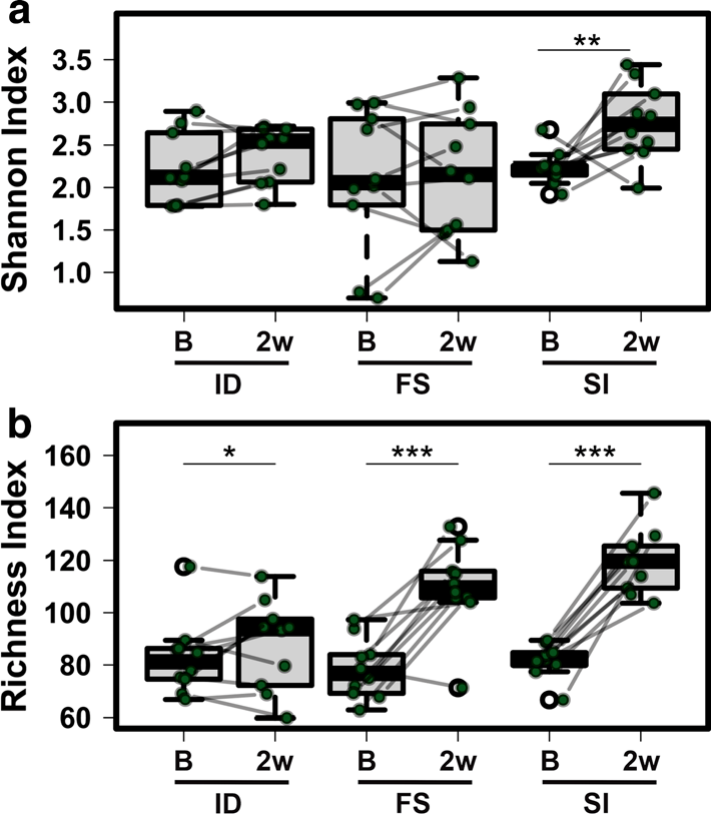
Changes in Shannon diversity and richness in the gut microbiota before and after iron supplementation. Each dot represents the Shannon **a** index or richness **b** of a mouse at baseline (B) or 2 weeks after iron supplementation (2w). A line connects the data points for each mouse before and after supplementation. Paired data points are indicated with green circles and outliers with white circles. *ID* iron-deficient, *FS* ferrous sulfate, *SI* Sucrosomial® iron. *P < 0.05, ** *P* < 0.01, ***P < 0.001

A mixed effect regression analysis was then carried out to analyse changes in alpha diversity between the two time points for each diet. The mixed effect model was built with diversity indexes (Shannon or richness) as the outcome, mice and cage as the random effects, diet as the independent variable and time as a fixed effect. All three diets had a significant increase in richness at the second time point (Mixed effect regression, ID: *P* = 0.012, FS: *P* < 0.001, SI: *P* < 0.001), with the iron-rich diets having the strongest increase followed by the iron deficient group (Fig. 4). Shannon diversity was lowest in ferrous sulfate treated mice and the highest in animals fed the Sucrosomial® iron containing diet at the second time-point. A significant change in Shannon diversity between the time-points was only measured in mice on Sucrosomial® iron containing diet (Mixed effect regression, SI: *P* < 0.006).

### Changes in microbial taxa between time-points and diets

**Fig. 5:**
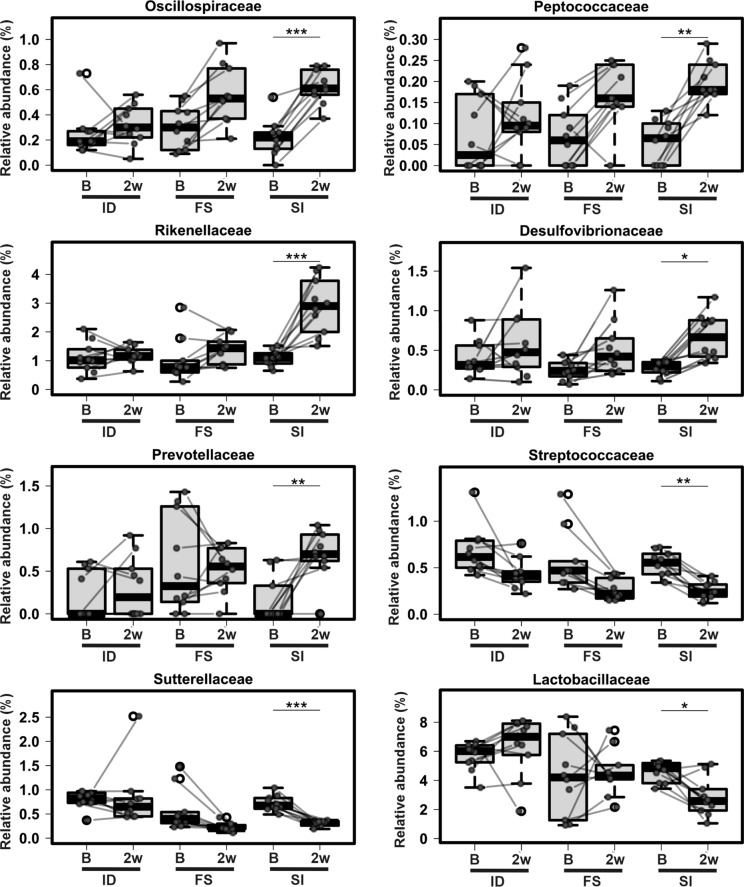
Longitudinal abundance of families in the gut microbiota at baseline and after iron supplementation. A linear mixed effects analysis with Bonferroni adjustment was performed and the abundance of selected bacterial families at baseline (B) or 2 weeks after iron supplementation (2w) is shown. A line connects the data points for each mouse before and after supplementation. Paired data points are indicated with green circles and outliers with white circles. *ID* iron-deficient, *FS* ferrous sulfate, *SI* Sucrosomial® iron. *P < 0.05, ** *P* < 0.01, ***P < 0.001

A mixed effects regression model was used to identify taxa at the phylum, family and Amplicon Single Variant (ASV) levels that differed between time points and within each diet group (Fig. 5, Supplementary Fig. S2 and S3). At the phylum level, Proteobacteria were significantly decreased in the Sucrosomial® iron treated animals (adjusted *P* < 0.001), but not in either the iron deficient or ferrous sulfate groups (Supplementary Fig. S2). Also, the phylum Desulfobacterota (adjusted *P* = 0.004) and its family *Desulfovibrionaceae* (adjusted *P* = 0.02) were increased significantly in the Sucrosomial® iron group, while the changes in the ferrous sulfate and iron deficient groups were not significant (*P* > 0.1). The phylum Bacteroidota was significantly increased in mice fed the iron deficient diet and decreased in those fed the ferrous sulfate containing diet (adjusted *P* < 0.05), but was overall unchanged in the Sucrosomial® iron group.

*Ruminococcaceae* and *Lachnospiraceae* (both phylum Firmicutes) increased at the family level following both Sucrosomial® iron (adjusted *P* < 0.001) and ferrous sulfate treatment (adjusted *P* < 0.05) (Supplementary Fig. S2). *Tannerellaceae* decreased in those animals on the iron supplemented diets, but increased in iron deficient mice (adjusted *P* < 0.05). *Oscillospiraceae*, *Peptococcaceae*, *Rikenellaceae*, *Desulfovibrionaceae* and *Prevotellaceae* increased, whereas *Streptococcaceae*, *Sutterellaceae* and *Lactobacillaceae* decreased significantly, but only in those animals on the Sucrosomial® iron diet following *P* value correction (adjusted *P* < 0.05, Fig. 5).

Several ASVs increased in the group fed the Sucrosomial® iron containing diet including *Lachnospiraceae* spp, *Rikenellaceae* RC9, *Faecalibaculum*, *Colidextribacter* and *Oscillibacter* (Supplementary Fig. S3). The ASV assigned to *Tuzzerella* increased in mice fed either iron rich diet. In contrast, ASVs classified as *Parasutterella* and *Tannerellacea* decreased in mice on the Sucrosomial® iron diet but remained unchanged in iron deficient and ferrous sulfate treated mice (adjusted *P* = 1). *Mucispirillum schaedleri* increased in iron-supplemented mice but did not reach significance following *P* value correction (SI: *P* < 0.05, adjusted *P* = 1, FS: *P* = 0.07). No change was seen in the ASVs assigned to *Escherichia* and *Shigella* species although the relative abundance was very low.

### Associations between the gut microbial diversity and iron indices

Regression analysis was applied to study associations between alpha diversity (Shannon or richness) and iron indices (Supplementary Fig. S4). A moderate positive association was observed between richness and mean cell volume (Pearson correlation, *P* < 0.001, r = 0.64). Moreover, a weak positive association was measured between richness and serum iron levels (Pearson correlation, *P* < 0.001, r = 0.589) and between richness and transferrin saturation (Pearson correlation, *P* = 0.002, r = 0.55).

## Discussion

Oral iron supplements are commonly used to treat iron deficiency and IDA. However, supplements, such as ferrous sulfate, are associated with gastrointestinal side effects such as nausea and abdominal pain, which can cause up to 50% of patients to discontinue treatment (Tolkien et al. 2015). These iron supplements can also affect the intestinal microbiome, leading to an increase in intestinal pathogens and a decrease in the abundance of beneficial bacteria in response to oral iron in some cases (Jaeggi et al. 2015; Paganini et al. 2017; Zimmermann et al. 2010). This likely reflects the relatively low fractional absorption of iron from supplements. For example, in a study of 21 iron deficient women given a daily dose 60 mg of iron as ferrous sulfate for fourteen days, an average of 10 mg was absorbed into the body each day, leaving 50 mg of unabsorbed iron in the lumen of the intestine (Stoffel et al. 2017). To put this in perspective, the total iron intake from the typical western diet is approximately 9–15 mg/day (Beck et al. 2014; Han et al. 2019). Therefore, iron supplements dramatically increase the amount of iron available to the intestinal microbiome. As the fractional absorption of iron supplements is generally low (Stoffel et al. 2017), novel iron formulations are required that reduce the impact of iron remaining in the lumen on the intestinal microbiome.

In the current study, we treated mice with iron as either Sucrosomial® iron or ferrous sulfate for two weeks. As both diets contained 50 mg iron/kg and animals in both treatment groups had the same iron status at the end of the study, it can be assumed that similar amounts of iron from each supplement remained in the lumen of the intestine. Therefore, any changes seen in the microbial composition of the feces should reflect the formulation of iron used. Our results suggest that oral Sucrosomial® iron has a beneficial effect on the intestinal microbiome when compared to ferrous sulfate. While both iron containing diets resulted in an increase in microbial richness over the course of the study, only the Sucrosomial® iron containing diet led to an increase in the Shannon index. In fact, Shannon diversity was lowest in mice fed the ferrous iron containing diet and highest in those on the Sucrosomial® iron containing diet. This indicates that mice consuming Sucrosomial® iron had a more diverse, and potentially healthier (Kriss et al. 2018), microbiome when compared to those on the ferrous sulfate containing or iron deficient diets.

The abundance of the phylum Proteobacteria, which contains many enteric pathobionts such as *Escherichia*, *Enterobacter* and *Salmonella*, was significantly decreased in the Sucrosomial® iron group while remaining unchanged in the iron deficient and ferrous sulfate fed mice. This suggests that Sucrosomial® iron may be a safer option for treating iron deficiency, as previous studies in infants and children have shown an increase the prevalence of Proteobacteria, including potentially pathogenic groups such as *Escherichia* and *Shigella* (Jaeggi et al. 2015; Zimmermann et al. 2010), following the use of conventional iron supplements. While no significant change in the prevalence of *Escherichia* or *Shigella* was observed in any of the groups in the current study, the relative abundance of each genus was very low. This agrees with previous findings in human trials showing that these groups only increase in prevalence in response to iron supplementation if the baseline pathogen burden is high (Dostal et al. 2014).

The most dominant genus within the phylum Proteobacteria was *Parasutterella*, which decreased significantly in the Sucrosomial® iron group. While *Parasutterella* is part of the core gut microbiota (Ju et al. 2019), it is associated with chronic intestinal inflammation (Chen et al. 2018) and is increased in the submucosal tissue of patients with advanced inflammatory bowel disease (Chiodini et al. 2015). In contrast, short chain fatty acid (SCFA) producing bacteria, including species of *Rikenellaceae*,(Graf 2014), *Lachnospiraceae* (Vacca et al. 2020), *Faecalibaculum* (Zagato et al. 2020), *Oscillibacter* (Iino et al. 2007) were more abundant in mice fed the Sucrosomial® iron containing diet than in those animals fed the ferrous sulfate containing or iron deficient diets. SCFAs are produced by the anaerobic fermentation of indigestible polysaccharides, predominantly by the microbes in the colon, and have been implicated in various aspects of health and disease, including the regulation of whole body energy metabolism (Li et al. 2018; Mollica et al. 2017) and the development of behavioural and neurological pathologies such as Alzheimer’s Disease and depression (Silva et al. 2020). SCFAs also have anti-inflammatory effects directly in the colon itself, and patients with inflammatory bowel disease often show reduced levels of both SCFAs and SCFA-producing bacteria (Parada Venegas et al. 2019). Interestingly, a recent study conducted in mildly anemic IBD patients showed that oral Sucrosomial® iron was not only well tolerated and effective at treating the anemia, but was also able to improve IBD activity scores (Abbati et al. 2019). It should be noted that this was a small, open label study with all participants receiving Sucrosomial® iron, and larger blinded studies are required to confirm these findings. However, the results from the current study suggest that the improvements in IBD symptoms reported previously may be due to favorable changes in the intestinal microbiome following treatment with Sucrosomial® iron.

The increase in *Faecalibaculum* in Sucrosomial® iron treated mice also has other potential implications for intestinal health. A recent study has demonstrated that SCFAs produced by *Faecalibaculum rodentium* and its human homolog *Holdemanella biformis* protect against the growth of intestinal tumors (Zagato et al. 2020). This could be of particular benefit in IBD patients who are more at risk of developing gastrointestinal malignancies (Axelrad et al. 2016), particularly as several studies in rodents show that oral ferrous iron can promote intestinal tumor development (Radulescu et al. 2012; Seril et al. 2002).

How Sucrosomial® iron induces these potentially beneficial changes to the intestinal microbiome when compared with ferrous sulfate is unclear. The diets contained equal amounts of iron and the body weights of the mice were not significantly different, which suggests that the iron consumed was relatively consistent between the groups. Sucrosomial® iron contains ferric pyrophosphate and it is possible that this form of iron is not as bioavailable as ferrous sulfate for certain bacteria. However, this is unlikely, as most bacteria produce siderophores that are powerful chelators of ferric iron (Page 2019), the most prevalent form of iron in the environment. The increase in bacterial diversity associated with Sucrosomial® iron treatment also suggests that this formulation can be utilised by the microbiome. Microbial diversity decreases when iron in the intestinal lumen is scarce (Pereira et al. 2015) and is often associated with increases in *Lactobacillus* abundance (Dostal et al. 2012), as this genus represents one of the few groups of organisms that do not require iron for survival (Bruyneel et al. 1989; Weinberg 1997). As seen in the current study, the relative abundance of *Lactobacillus* species declines when iron in the gut lumen is increased (La Carpia et al. 2019; Zimmermann et al. 2010), as iron-dependent bacteria are able to more rapidly proliferate. These results imply that Sucrosomial® iron is available to bacteria in the intestine. Sucrosomial® iron is made of a ferric pyrophosphate core surrounded by a phospholipid matrix coated with the sucrose ester of fatty acids, sucrester (Gomez-Ramirez et al. 2018). Some sucrose esters have been shown to have antimicrobial properties (Petrova et al. 2017), however, whether this applies to sucrester is unclear and further research is needed to explain the beneficial effects of Sucrosomial® iron on the intestinal microbiome.

In conclusion, the changes in the intestinal microbiome induced by a Sucrosomial® iron containing diet are likely favourable, with an increase in microbial diversity when compared to a ferrous sulfate containing diet or an iron deficient diet. An increase is microbial diversity is generally considered to reflect a healthier microbiome (Kriss et al. 2018). Most commonly, an increase in diversity is associated with a decrease in pathogenic bacteria belonging to the phylum Proteobacteria. In agreement with this, we have observed a decrease in this phylum in the microbiome of mice fed the Sucrosomial® iron containing diet. This suggests that Sucrosomial® iron would have benefits over other commonly used iron supplements, particularly when used in areas where the risk of gastrointestinal infections is high. Sucrosomial® iron also favoured the growth of beneficial bacteria known to produce SCFAs. While the results presented where carried out under controlled laboratory conditions and may not reflect the microbial responses in general human populations taking Sucrosomial® iron supplements, the data suggest that Sucrosomial® iron has beneficial effects on the intestinal microbiome when compared to ferrous sulfate in a mouse model.

## Supplementary Information

Below is the link to the electronic supplementary material.

Supplementary file1 (docx 1503 KB)

## Data Availability

The data that support the findings of this study have been uploaded to the NCBI Sequence Read Archive at http://www.ncbi.nlm.nih.gov/bioproject/754104 with the project accession number PRJNA754104.
