# Supplementary material for: Supplementation with Sucrosomial® iron leads to favourable changes in the intestinal microbiome when compared to ferrous sulfate in mice
Source: Biometals. 2021 Oct 25;35(1):27–38. doi: 10.1007/s10534-021-00348-3 (PMC8803775; doi:10.1007/s10534-021-00348-3)
Supplement: Supplementary file 1 — Supplementary file1 (docx 1503 KB) [file 10534_2021_348_MOESM1_ESM.docx]

**Supplementary data for**

**Supplementation with Sucrosomial^®^ iron leads to favourable changes in the intestinal microbiome when compared to ferrous sulfate in mice.**

Martha Zakrzewski^1^, Sarah J. Wilkins^2^, Sheridan L. Helman^3,4^, Elisa Brilli^5^, Germano Tarantino^5^, Gregory J. Anderson^2,6^, and David M. Frazer^3,7,8^.

^1^Medical Genomics, QIMR Berghofer Medical Research Institute, Herston, Australia, ^2^Iron Metabolism Laboratory, QIMR Berghofer Medical Research Institute, Herston, Australia, ^3^Molecular Nutrition Laboratory, QIMR Berghofer Medical Research Institute, Herston, Australia, ^4^Faculty of Medicine, The University of Queensland, St Lucia, Australia, ^5^R&D Department, PharmaNutra S.p.A, Pisa, Italy, ^6^School of Chemistry and Molecular Bioscience, The University of Queensland, St Lucia, Australia, ^7^School of Biomedical Sciences, The University of Queensland, St Lucia, Australia, ^8^School of Biomedical Sciences, the Queensland University of Technology, Gardens Point, Australia.

Corresponding Author: David M Frazer, Molecular Nutrition Laboratory, QIMR Berghofer Medical Research Institute, Locked Bag 2000, Royal Brisbane Hospital, Queensland, 4029, Australia; phone: +61-7-3845-3063; email: [david.frazer@qimrberghofer.edu.au](mailto:david.frazer@qimrberghofer.edu.au).


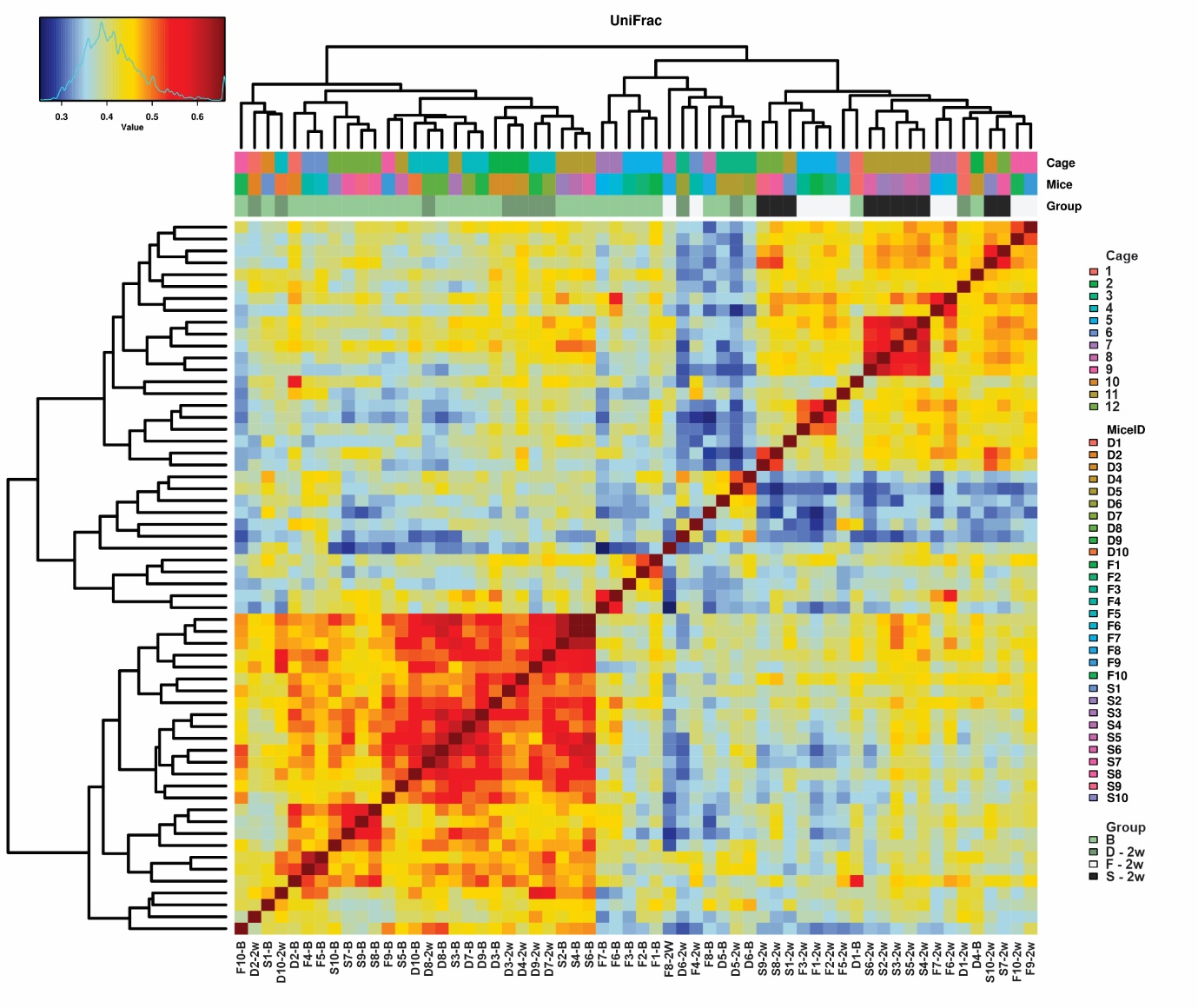


**Supplementary Fig. S1 Hierarchical clustering dendrogram and heatmap of gut microbiota samples.** Dendrograms of the gut microbiota indicate the relationship between the samples based on unweighted unifrac distances. Samples are colored by cage number, mouse identification number and diet group (legend on the right). The heatmap depicts the similarity between each sample combination. The color scale of the heatmap is displayed in the upper left corner of the figure (red: high similarity, blue low similarity). Samples from mice fed the iron-deficient diet at baseline and at the end of the study form a cluster. Cage refers to the individual cage that mice were housed in. Mice refers to the individual mice within each treatment group. B – mice at baseline after 2 weeks on an iron deficient diet; D – mice fed the iron deficient diet; F – mice fed the ferrous sulfate containing diet; S – mice fed the Sucrosomial^®^ iron containing diet; 2w – Mice maintained for a further two weeks from baseline on the diet indicated.


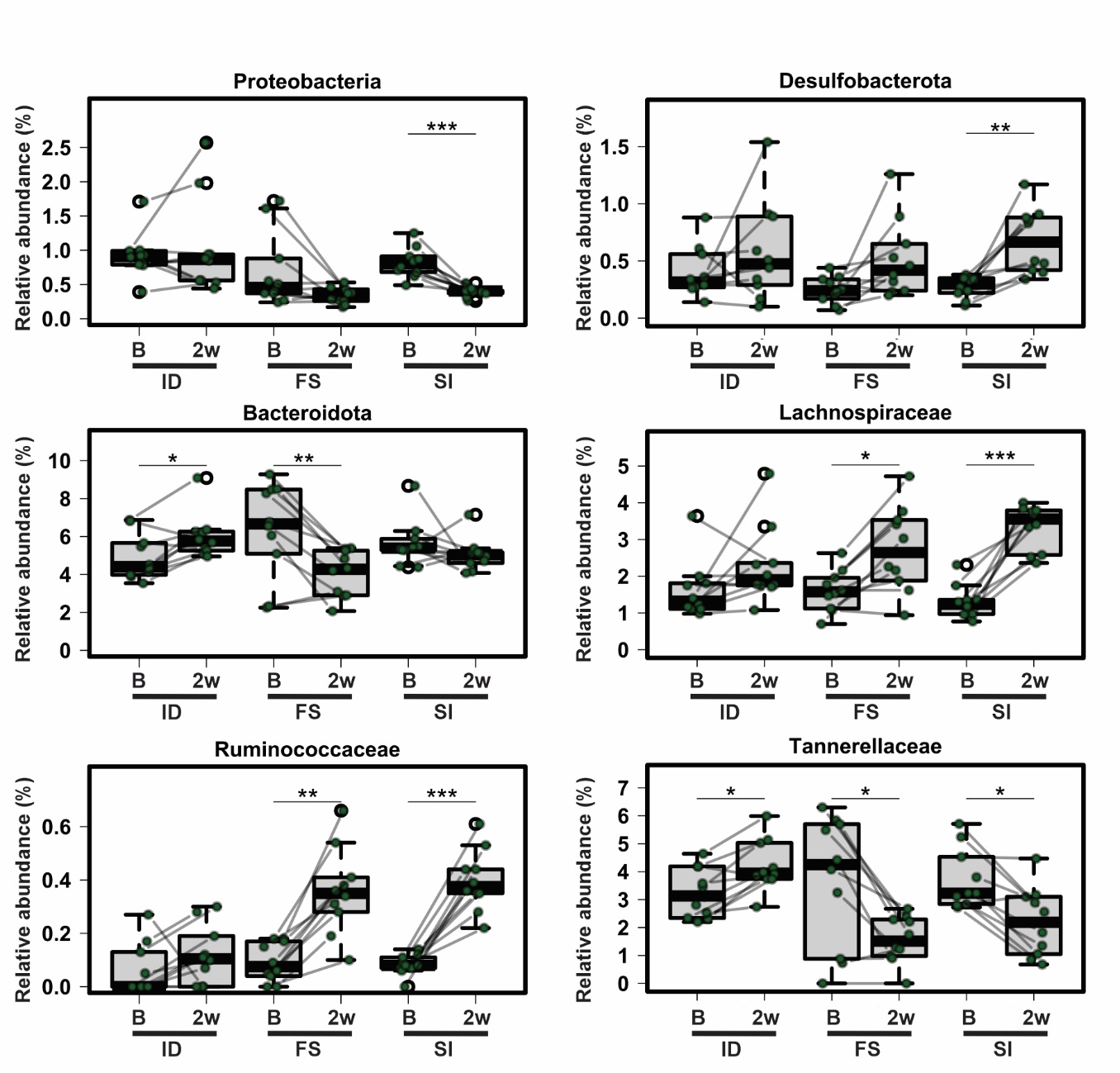


**Supplementary Fig. S2 Changes of taxa at the phylum and family levels between different time points within each diet.** Boxplots showing significant changes in the phyla Proteobacteria, Desulfobacterota and Bacteroidota and families Lachnospiraceae, Ruminococcaceae and Tannerellaceae between baseline (B) and 2 weeks after diet supplementation (2w). Diets were iron-deficient (ID), ferrous sulfate (FS) and Sucrosomial^®^ iron (SI). A line connects the data points for each mouse before and after supplementation. Paired data points are indicated with green circles and outliers with white circles. Significant taxa were identified using mixed effect regression models and Bonferroni correction. **P* < 0.05, ** *P* < 0.01, ****P* < 0.001.


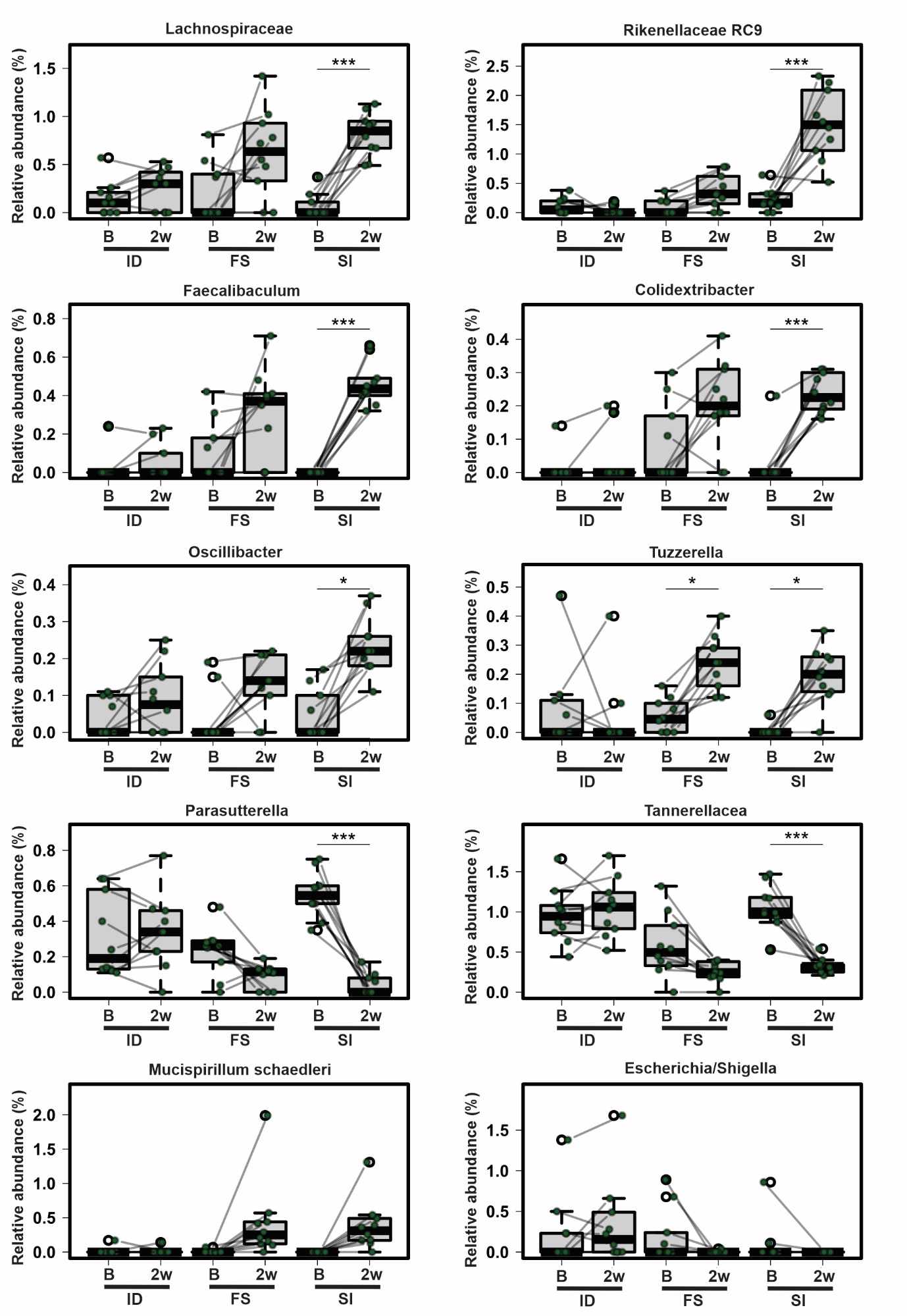


**Supplementary Fig. S3 Changes of ASVs between different time points within each diet.** Boxplot showing changes in ASVs for each diet group between baseline (B) and 2 weeks after diet supplementation (2w). Diets were iron-deficient (ID), ferrous sulfate (FS) and Sucrosomial® iron (SI). A line connects the data points for each mouse before and after supplementation. Paired data points are indicated with green circles and outliers with white circles. Significant taxa were identified using mixed effect regression models and Bonferroni correction. **P* < 0.05, ** *P* < 0.01, ****P* < 0.001.


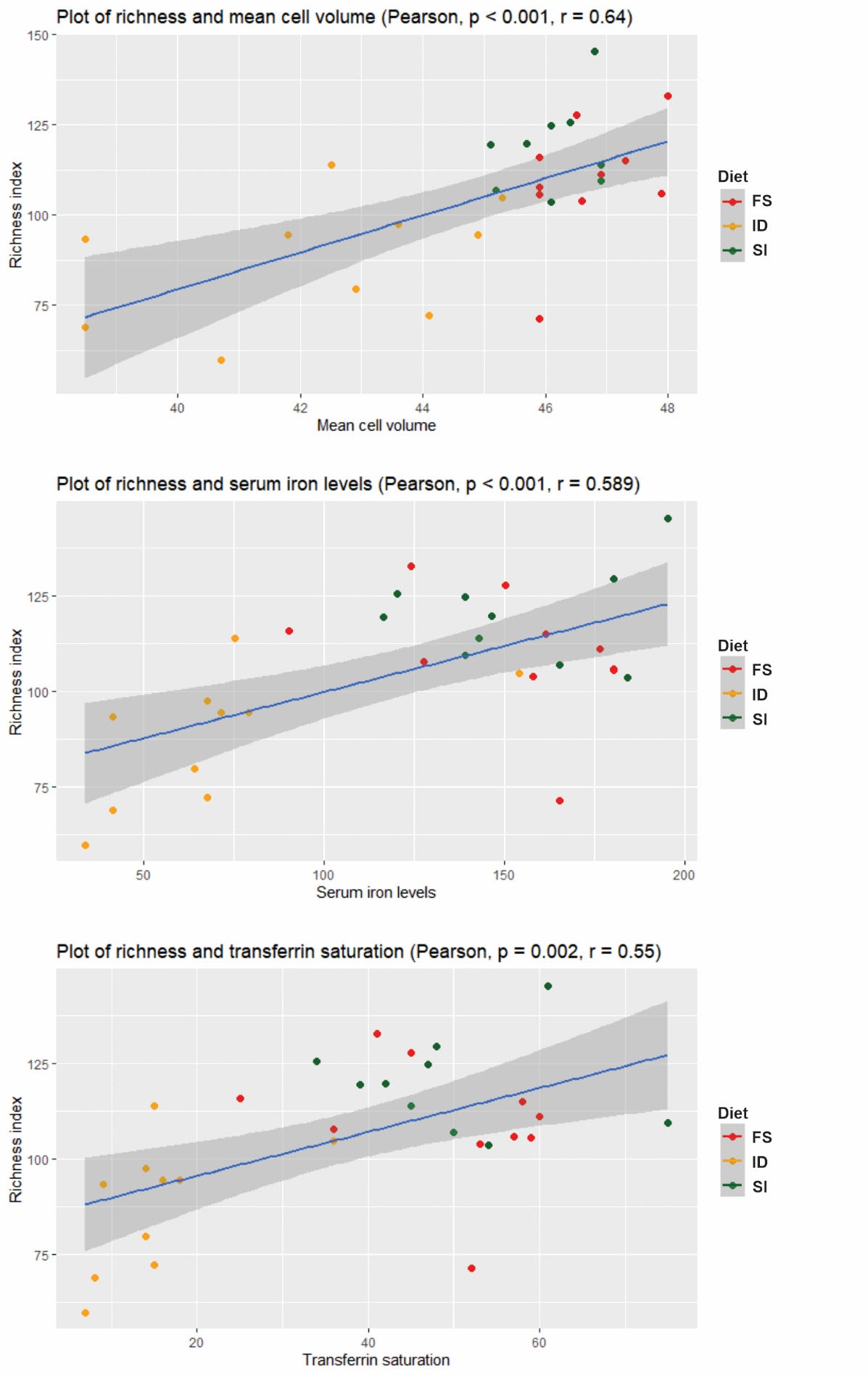


**Supplementary Fig. S4 Correlation analysis between richness and mean cell volume, serum iron levels and transferrin saturation.** The richness index and iron parameters are plotted for each mouse at the second time point (after 2 weeks on specific diets). The data points are colored according to the mouse diet. Correlation was measured using Pearson correlation test. ID – iron-deficient, FS – ferrous sulfate, SI – Sucrosomial^®^ iron.
